# Water Intake, Dietary Acid Load, and Body Composition in Aging Females

**DOI:** 10.3390/nu17111808

**Published:** 2025-05-26

**Authors:** Olga Januszko, Agata Białecka-Dębek

**Affiliations:** Department of Human Nutrition, Institute of Human Nutrition Sciences, Warsaw University of Life Sciences—SGGW, 02-776 Warsaw, Poland; olga_januszko@sggw.edu.pl

**Keywords:** women’s health, older women, nutrition, dietary pattern, water intake, dietary acid load, body composition

## Abstract

**Background/Objectives:** Aging is a natural, gradual, and irreversible process associated with disruptions in homeostasis, causing several unfavorable changes, including changes in body composition. Some studies show that higher water intake can be related to a healthier body composition, but studies in older adults are rare. The aim of this study is to assess the relationship between water intake, dietary acid load, and body composition, and also to assess the interrelationship of these two factors with body composition in older women. **Methods**: This cross-sectional study involved 195 independently living women aged 65–84. Body composition was assessed using fan-beamed dual-energy X-ray absorptiometry (DXA). Potential renal acid load (PRAL) and water intake were evaluated using the 3-day food record method. **Results:** Higher water intake was associated with significantly lower android (*p* = 0.004), gynoid (*p* < 0.001), and total body fat mass (*p* = 0.005), as well as higher lean body mass (*p* = 0.021). Among the assessed anthropometric indicators, only for the appendicular lean mass (ALM) was there a significant difference between the “low-PRAL diet” compared to the “high-PRAL diet” (6.2 ± 0.8 vs. 6.0 ± 0.9 kg/m^2^; *p* = 0.045). A “low-PRAL diet” was characterized by a significantly lower energy value, lower protein intake, and a higher plant-to-animal protein ratio than a “high-PRAL diet”. **Conclusions:** A more acidifying diet pattern appeared to have an adverse effect on lean body mass in older women. The ratio of plant to animal protein may be crucial in this relationship.

## 1. Introduction

Aging is a natural, gradual, and irreversible process associated with disruptions in homeostasis, causing several unfavorable changes, including changes in body composition. This can result in a decrease in lean mass and an increase in fat mass [1]. Epidemiological studies indicate that the percentage of overweight/obese adults increases with age [2]. Abdominal obesity is unfavorable because it is a significant risk factor for systemic inflammation, insulin resistance, and metabolic syndrome [3]. On the other hand, as individuals age, there is a progressive loss of muscle mass and strength known as sarcopenia, which can coexist with obesity [4,5]. There is no clear definition of the mechanisms that lead to sarcopenia. Age, sex, level of physical activity, and dietary habits have been well described as risk factors for sarcopenia [6]. It has been demonstrated that the incidence of sarcopenia in elderly populations is significantly higher in women than in men, with the risk increasing markedly with advancing age in both sexes [7]. It is generally observed that muscle mass reaches its peak at approximately 30 years of age, after which it gradually declines. By age 70, there is often a 20–40% decrease in muscle mass, which can lead to sarcopenia [8].

Aging is also associated with decreased muscle water content [9]. There is a consensus that water is essential for the maintenance of life. It is necessary for many biological processes. These include thermoregulation, regulating blood pressure, facilitating biochemical reactions, and transporting nutrients into and waste out of cells. The body’s water content tends to fluctuate gradually throughout life [10]. The European Food Safety Authority (EFSA) recommends 2.0 L of water daily for women and 2.5 L/day for men [11]. EFSA includes all water consumed from different sources, including drinking water, beverages, and foods with moisture content. Knowing that approximately 20% of water comes from food and 80% from fluids, the European Society for Clinical Nutrition and Metabolism (ESPEN) recommends fluid intake for older adults: 1.6 L/day for women and 2.0 L/day for men [12]. The amount of water demand can vary quite significantly among different groups of people. It depends on many factors, primarily gender, age, and the degree of physical activity [11]. According to the literature, the average fluid intake in older adults varies between 311 and 2390 mL/day [13]. Several studies have demonstrated a correlation between reduced fluid intake and an increased risk of developing various diseases and health conditions. These include urolithiasis, bladder fibrosis, constipation, urinary tract infections, asthma, dental disease, cardiovascular disease, diabetes, glaucoma, and cancers of the bladder and colon [14]. Some studies show that higher water intake can be related to a healthier body composition [15], but studies in older adults are rare. To the best of our knowledge, there are only a few studies that evaluate the relationship between water intake and body composition. A significant association between total water intake and lean body mass was found in a study of healthy children and adolescents [16]. Moreover, studies conducted among healthy adolescents and young adults have demonstrated that elevated water intake positively correlates with body water content and inversely correlates with body mass index (BMI) and fat body mass [15,17]. Additionally, a significant correlation was observed between total water intake and body fat and body water content percentage in a cohort of Spanish aeronautical military personnel [18]. There are limited data on this association in older adults. We hypothesize that, similar to younger age groups, higher water consumption will have a positive effect on body composition [15].

A substantial body of research has examined the impact of macronutrient and micronutrient deficiencies or excesses on various aspects of body composition and functionality in the context of aging [8]. Dietary composition is of great consequence in maintaining the body’s acid–base equilibrium. Dietary acid load (DAL) can be calculated using two methods, including potential renal acid load (PRAL) and net endogenous acid production (NEAP), which reflect the balance between acid-producing and base-producing foods in the diet. The PRAL can be estimated based on the content of food components such as protein, potassium, phosphorus, calcium, and magnesium [19]. Foods like fruits and vegetables, rich in calcium, magnesium, and potassium, have an alkalizing effect. Foods such as grains, meat, cheese, and eggs produce more sulfuric and phosphoric acids, raising the body’s acid load. This is predominantly attributable to their elevated content of sulfur-containing amino acids (e.g., lysine and arginine), which serve as principal dietary acid precursors. Milk and fats are neutral in this regard [20]. A relationship has been shown between high PRAL values and numerous diseases, including diabetes, osteoporosis, cardiovascular diseases, cancer, and kidney diseases [19]. Additionally, recent research has linked higher DAL to depression, anxiety, and sleep disorders [21,22,23]. Higher PRAL and NEAP were associated with a higher incidence of frailty [24]. A growing number of studies in various populations worldwide suggest that these nutritional factors are closely related to bone mineral density (BMD) [25,26,27]. Furthermore, high PRAL will potentially adversely affect body composition by reducing lean body mass [28,29,30,31]. Several studies have indicated a positive correlation between high DAL and various anthropometric measurements, including an increased waist-to-height ratio, larger hip and neck circumference, higher fat mass, and lower fat-free mass. However, it is notable that most of these studies have been conducted with younger age groups, including children, adolescents, and students [32,33,34].

This study aims to assess the relationship between water intake, dietary acid load, and body composition, but the novelty of this study is to evaluate the interrelationship of these two factors with body composition in older women. Given the variability in age-related changes in muscle and fat distribution, their correlation with health outcomes between genders [35], and the significant dietary differences between men and women [36,37], this study was limited to women.

## 2. Materials and Methods

**Figure 1:**
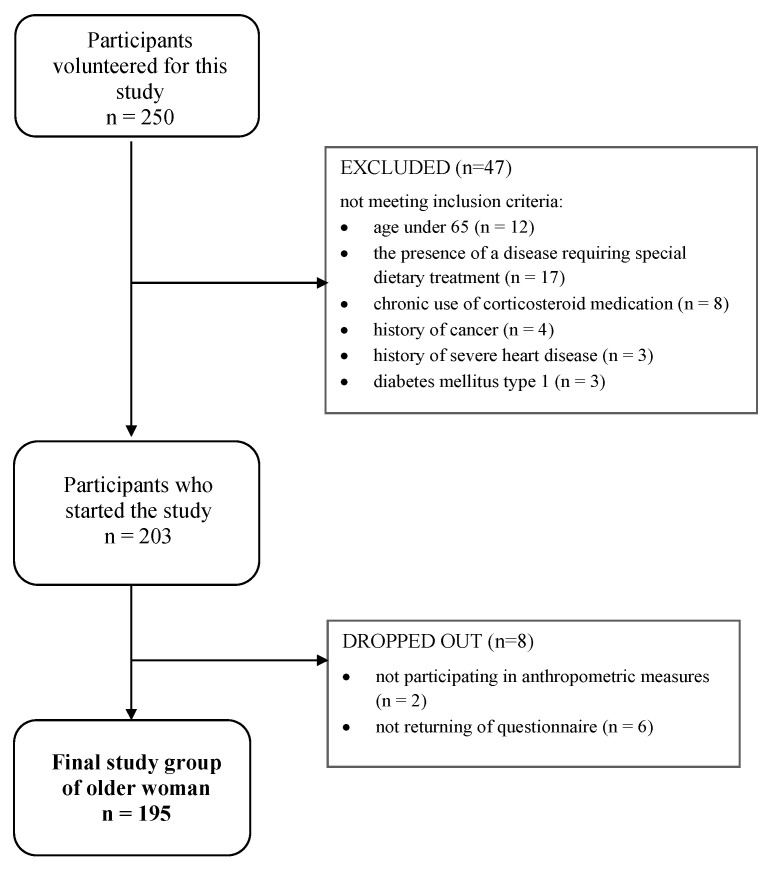
Flow chart of the study population.

The cross-sectional study involved 195 apparently healthy, independently living women aged 65–84. Written informed consent was collected from all participants before inclusion in the study by the Declaration of Helsinki (The World Medical Association Inc., Ferney-Voltaire, France, 2018. “THE HELSINKI DECLARATION Ethical Principles for Medical Research Involving Human Subjects”). The Bioethics Committee of the Ethical Committee of the Warsaw University of Life Sciences approved this study in 2019 (Resolution No. 50p/2019). Sampling for this study was voluntary, and participants were those who volunteered to take part in this study. We used screening questionnaires to verify that none of the exclusion criteria were present. The inclusion and exclusion criteria of eligible participants can be found in Table 1. A total of 250 participants volunteered for this study; however, 47 participants were found to not meet the inclusion criteria, and 8 dropped out during this study (Figure 1).

Data were collected using a questionnaire that included the following sections: (1) sociodemographic characteristics, (2) health and lifestyle status, (3) self-reported level of nutritional knowledge and dietary habits, and (4) dietary supplements used in the six months before this study. The sociodemographic questions looked at data on age, marital status, level of education, and place of residence. Health and lifestyle questions provided data on self-reported health status, occurrence of chronic diseases, hospitalization within the last year, cigarette smoking, alcohol drinking, and self-reported physical activity levels. To assist respondents in selecting an appropriate physical activity category, each category provided examples of types of exercise and the number of hours spent weekly on these activities.

Data on food consumption were collected using the 3-day food record method, covering two non-consecutive weekdays and one weekend day. Unlike food frequency questionnaires and 24 h recalls, food records minimize reliance on memory, as older adults recorded their food intake as they consumed it [38]. According to the literature, the dietary record should be long enough to provide reliable information on usual intake, but balanced against the risk of poor adherence if the record is too long. Reminder periods longer than 4 consecutive days were considered generally unsatisfactory, so a 3-day record was adopted [39]. Participants were instructed to provide detailed information about consumed foods and beverages and determine portion sizes (using kitchen scales or household measures). Participants recorded their dietary intake continuously over the three assessment days, completing a provided blank diary at home. Researchers reviewed the returned questionnaires. Inaccurate or missing portion size information was corrected with respondents using a product and dish photo album [40].

For the estimation of energy, water, and nutrient content of the respondents’ diets, the software “DIETA 6.0” (Polish National Institute of Public Health—PZH), based on the Polish tables of food composition and nutritional values, was used [41,42]. For total water intake, “DIETA 6.0” analyzed the water content per serving of each food or ingredient, providing total water intake from water in all beverages and foods. According to the literature, data collected using the 3- or 4-day diary method are recommended to assess water intake [43]. In addition, the ratio of plant to animal protein (PP ratio) was calculated.

The following formula was used to calculate PRAL scores [44]:

PRAL (mEq/d) = 0.4888 × protein intake (g/d) + 0.0366 × phosphorus (mg/d) − 0.0205 × potassium (mg/d) − 0.0125 × calcium (mg/d) − 0.0263 × magnesium (mg/d)

Based on the previous literature [44], a value of PRAL < 0 indicates base-forming potential and PRAL > 0 acid-forming potential.

Body composition was assessed using fan-beamed dual-energy X-ray absorptiometry (DXA)(Lunar Prodigy, GE Healthcare, Madison, WI, USA—enCORETM 2011 software version 13.6) using standardized protocols [45,46]. Trained technicians performed DXA scans following state-of-the-art technology and manufacturer recommendations. The following measurements were obtained: total body fat (TBF), lean mass, and fat mass in the android and gynoid regions. Appendicular lean mass (ALM, kg/m^2^) was calculated using the sum of lean mass in both upper and lower limbs from DXA to height square.

The participants’ height (in centimeters) was measured using a Harpenden stadiometer, while their weight (in kilograms) was recorded on a standard balance beam or digital scale. During this process, the participants were asked to take off their shoes and put on light clothing. Body mass index (BMI) was calculated by dividing weight (in kilograms) by height (in meters) squared (kg/m^2^).

Hand grip strength (HGS) was measured using a Smedley dynamometer (Scandidact, Odder, Denmark, ref. no. 281128). Subjects were asked to maintain an upright posture, with the arm at a right angle, and to hold the dynamometer to perform the strength test. The test was repeated three times with both hands, with a 60 s rest between each measurement. The maximum absolute hand grip strength was the sum of the dominant hand’s mean highest peak hand grip strength.

Data were analyzed using Statistica software version 13.3 (TIBCO Software Inc., Palo Alto, CA, USA). The results with *p*-values ≤ 0.05 were considered statistically significant. Individuals with missing data were not included in the analysis. The study group was divided into tertiles of water consumption and tertiles of the PRAL. Data were presented as means and standard deviations (mean ± SD) for quantitative variables and as frequencies and percentages (n and %) for categorical variables. Comparisons of parameters between 3 groups were tested by 1-factor ANOVA for the continuous variable or Pearson’s chi-square test for categorical variables. The Shapiro–Wilk test was employed to assess the normal distribution of the quantitative variables. Comparisons of parameters between 3 groups were tested by 1-factor ANOVA for variables with normal distribution or the Kruskal–Wallis test for variables with non-normal distribution. Spearman correlation coefficients were used to determine associations between water intake, PRAL, and body composition. Partial correlations were assessed to take into account for the potential effect of the following parameters on the observed associations: age (years, continuous), physical activity (medium or high, low), living alone (yes, no), smoking (ever, never), alcohol consumption (ever, never), and medication use (no, yes).

Dietary acid load was derived using cluster analysis. The input variables were the diet’s energy value, protein content, plant-to-animal protein ratio, and PRAL. All the input variables were standardized to obtain a mean equal to 0 and a standard deviation equal to 1. They were expressed as Z-score values. Participants were grouped based on Euclidean distances (a posteriori analysis) using the K-means clustering method. The following cluster analysis criteria were adopted: grouped by cases, number of clusters: 2; initial cluster centers: selected observations to maximize cluster distances, number of iterations: 5. Finally, two clusters were conducted and marked as “low-PRAL diet” (Cluster 1) and “high-PRAL diet” (Cluster 2).

## 3. Results

Characteristics of the participants across tertiles of water intake and estimated PRAL are shown in Table 2. The average water consumption in the study group was 2441.1 ± 577.7 mL/day, and the PRAL value was 0.9 ± 14.3 mEq/day (Table 3). Participants with higher water intake had a statistically significantly higher number of years of education. For other sociodemographic and lifestyle variables, there were no significant differences between the tertiles of water consumption and the tertiles of PRAL.

### 3.1. Water Intake and Body Composition

The relationship between tertiles of water intake and anthropometric measurements, body composition, and dietary characteristics are shown in Table 3. Higher water intake was associated with significantly lower android, gynoid, and total body fat mass, as well as higher lean body mass. Higher water intake was statistically significantly associated with higher dietary fiber intake. There was no difference in energy and protein intake and PRAL value between water intake tertiles.

### 3.2. Dietary Acid Load and Body Composition

The relationship between tertiles of PRAL and anthropometric measurements, body composition, and dietary characteristics are shown in Table 4. Higher PRAL was associated with significantly higher energy and protein intake. There were no statistically significant differences in body composition parameters between PRAL tertiles.

Correlations between water intake and body composition are shown in Table 5. Total water intake was negatively correlated with android, gynoid, and total body fat mass, and positively correlated with lean body mass, even after partial correlation coefficients were adjusted for age, physical activity, living alone, smoking, alcohol consumption, and medication use. PRAL was negatively correlated with ALM, also after partial correlation coefficients (Table 5).

### 3.3. Cluster Analysis

Two dietary patterns emerged from the cluster analysis (Figure S1) and were marked as “low-PRAL diet” (Cluster 1) and “high-PRAL diet” (Cluster 2).

The characteristics of study participants according to cluster analysis are presented in the Supplementary Material (Table S1). The study group was divided using cluster analysis into two groups differing significantly regarding the diet’s energy value, protein content, plant-to-animal protein proportion, and PRAL (Table 6). Cluster 1 was characterized by a significantly lower PRAL and lower energy value of the diet, lower protein intake, and a higher ratio of plant to animal protein compared to Cluster 2. At the same time, water consumption did not differ significantly between the clusters. Among the assessed anthropometric indicators, there was only a significant difference between the clusters for the ALM. A higher value was observed in Cluster 1 compared to 2 (6.2 ± 0.8 vs. 6.0 ± 0.9 kg/m^2^; *p* = 0.045).

## 4. Discussion

The increasing number of older people in society is becoming a public health challenge. As the body ages, numerous unfavorable changes in its composition occur, including bone mass and muscle mass loss and an increase in fat mass [8]. Adverse changes in body composition, mainly an increase in adipose tissue in older people, are associated with poor physical performance. In contrast, lean mass percentage is associated with better physical performance [47].

In this study, higher water intake was associated with healthier body composition, including significantly lower android and gynoid fat mass and total body fat, as well as higher lean body mass. Research suggests that increased water intake is associated with weight loss in overweight and obese adults [48,49,50], mainly by reducing total energy intake [48,49,51]. Water intake is also related to the activity of the sympathetic nervous system, which increases daily energy expenditure and thermogenesis [52]. However, researchers emphasize that the long-term effects of drinking water on changes in body weight and body composition are unknown [48], and the evidence for an association between water consumption and body weight outcomes is inconclusive, mainly due to a lack of good-quality studies [53]. A meta-analysis of eight RCTs showed that interventions promoting water consumption may not significantly affect adiposity in overweight and obese individuals [54]. Only a few studies in this field have looked at older people. Water intake has been shown to reduce energy intake in obese [55] and non-obese older people [56].

Inadequately hydrated adults had higher BMIs and were more likely to be obese than hydrated adults in the nationally representative sample of the National Health and Nutrition Examination Survey (NHANES) [57]. Maffeis et al. observed that obese children were less hydrated than normal-weight children, which was also related to differences in water intake. Additionally, BMI correlated with hydration status [58]. Studies among healthy adolescents and young adults also suggest that higher water balance and intake are associated with healthier body composition, estimated by bioelectrical impedance analysis. Water intake was positively correlated with body water content and inversely with BMI and fat body mass [15,17]. Total water intake was also significantly related to the percentage of body fat and percentage of body water content in aeronautical military personnel from Spain [18]. Clayton et al. showed that total water intake was significantly related to lean mass in healthy children and adolescents [16].

There are limited data on this association in older adults. In a nationally representative sample of Portuguese older adults (≥65 years, n = 1315), being in the third tertile of urinary osmolality (the highest) was associated with a higher risk of obesity in men but not in women. These findings highlight the need for research to clarify the relationship between hydration and weight status in older adults [59].

Water intake has been shown to increase metabolic rate significantly [60] through an increase in sympathetic nerve activity and plasma norepinephrine levels [61]. Water intake is adequate for losing weight through two main mechanisms: reduced energy intake and increased fat oxidation [62]. Physiologically, increased water intake results in increased blood volume with a concomitant increase in proper atrial pressure. This leads to the release of atrial natriuretic peptide (ANP) [63]. Short-term intravenous administration of ANP is associated with a rapid increase in lipid oxidation [64]. Water intake reduces arginine vasopressin (AVP). AVP is the primary endocrine regulator of renal water excretion and appears to be a negative regulator of adipose tissue energy metabolism [65]. There may be bidirectional modulatory links between the AVP signal and gut microbiota composition [66]. Research has confirmed that the gut microbiome plays a vital role in the onset and development of obesity [67]. Some studies suggest drinking water may be necessary for shaping the human gut microbiome. However, little is known about this relationship [68]. It should be emphasized that the effect of chronic mild underhydration goes beyond promoting obesity. Extracellular dehydration may contribute to cardiovascular disease, diabetes, cancer, and Alzheimer’s disease [69]. As there is increasing evidence that inadequate water intake and underhydration may increase cardiometabolic risk [70], it seems even more important to assess the relationship between water intake and body composition in a group of older people, as studies in this population are rare.

Several studies found that elevated consumption of drinking water was associated with diet quality [71,72,73], healthier dietary patterns [74], better micronutrient adequacy [75], and lower energy intake [75]. This study indicates a relationship between high water intake and high fiber intake, which can suggest high consumption of vegetables and fruits high in water. High water intake could stem from both the large volume of water-rich foods consumed (vegetables, fruits) and the active consumption of beverages. A diet rich in plant-based foods with high water content is associated with generally higher water intake [76]. This relationship requires further investigation.

To our knowledge, no studies have evaluated the relationship between water consumption and diet acidifying potential. Our study observed no significant relationships between water intake and PRAL. The PRAL index value also did not correlate with anthropometric parameters. Among the assessed anthropometric indicators, only for the ALM was a significant difference between the “low-PRAL diet” (cluster 1) and “high-PRAL diet” (cluster 2). Although the difference in ALM (6.2 vs. 6.0 kg/m^2^) reaches statistical significance (*p* = 0.045), its clinical relevance is likely limited. Other studies have also confirmed these results. PRAL and NEAP were inversely related to skeletal muscle mass index among 390 overweight/obese women aged 18–64 [30]. In 2689 women aged 18–79 from the TwinsUK study, fat-free mass was positively associated with a more alkalinogenic dietary load [29]. The percentage of total lean body mass decreased significantly across PRAL quartiles only in women, but not in men, in a group of 243 seniors aged ≥ 60 years. In addition, the negative association was most pronounced in women with a low intake of protein (<1 g/kg of body weight per day) [31]. A 4-year prospective study of 3122 community-dwelling Chinese older adults aged 65 years and older provided evidence of a slower decline in appendicular skeletal muscle (ASM) mass with lower dietary acid load [28]. However, Storz points to differences in DAL values between populations and ethnic groups [77]. Further, older people are particularly vulnerable to acidification of the body from even a slightly alkaline diet, as measured by PRAL or NEAP indices, because kidney function deteriorates with age [78].

Protein intake is necessary to maintain muscle mass [79]. However, our results suggest that plant to animal protein ratio appears to be equally important in maintaining lean body mass. Direct evaluations of the effect of protein type on sarcopenia are limited. In studies of a middle-aged and older Chinese population, higher dietary protein intake, regardless of animal or plant source, was associated with more remarkable preservation of ALM and its index (ASMI) [80] and greater skeletal muscle mass [81]. On the other hand, higher intakes of plant protein, but not total and animal protein, were associated with less decline in muscle mass and walking speed in sarcopenia-free participants aged ≥ 65 years recruited from the Chinese community [82]. A lower risk of frailty was seen in women who consumed more plant protein and a higher risk in women who consumed more animal protein, regardless of total protein intake [83]. Some authors emphasize that sex and race may influence associations between protein intake and physical outcomes [84].

In this study, the consumption of protein, carbohydrates, and fats increased with higher PRAL, which is also confirmed by other studies [85]. However, a significantly higher fiber intake was shown in the group with high PRAL, contrary to results from previous studies. A statistically significant lower dietary fiber intake was found for high PRAL compared to low PRAL in a survey of 175 adults examining the relationship between dietary acid load and the severity of musculoskeletal pain [85]. Similarly, a large cross-sectional study of 29,683 people of different races/ethnicities found that an alkalizing diet (low PRAL) was significantly associated with more fiber than an acidifying diet (high PRAL) [86]. The total energy intake explains the differences in nutrient intake between the PRAL terciles and clusters. According to the literature, higher fiber intake is associated with better body composition. This is determined by a decrease in fat mass and an increase in lean mass [87]. Therefore, the higher fiber intake in the acidifying diet pattern (cluster 2) may have caused the body composition results to be non-significant.

A limitation of this study was the small number of participants and the recruitment of healthy people who volunteered to participate due to their willingness to include non-institutionalized people. Therefore, these were individuals without symptoms of muscle impairment, frailty syndrome, or sarcopenia. As a result, there was no comparison group, and it was impossible to provide a more detailed analysis of the relationship between hydration and PRAL and muscle mass and strength. This study is limited to women, which may limit the generalizability of the results. Future studies should include a more diverse population and a larger sample size to enhance external validity. This homogeneity in the study group may be a limitation of this study; on the other hand, it can be seen as an advantage because although the group was small, it was homogeneous in terms of age, education, physical, and social activity. Another strength was the research methods used, including an objective assessment of body composition by DXA and a method of assessing nutrition that does not rely on memory, such as food records. It should be remembered that all methods of dietary assessment based on self-recording of food consumption may be subject to error [88]. A limitation of this study is the lack of assessment of body water balance. Older persons, women, and obese individuals have lower total body water (TBW) because of their lower lean body mass [89]. Future studies should focus on a more accurate assessment of water intake and body hydration status, using recommended methods such as plasma osmolality. Another limitation of this study was its cross-sectional design, which makes it impossible to determine causality. Longitudinal studies should be carried out to explore these relationships further.

## 5. Conclusions

Our findings indicate that higher water intake was associated with healthier body composition in apparently healthy older women, a indicated by lower android, gynoid, and total body fat mass, as well as higher lean body mass. Although associations are reported, causality cannot be established due to the cross-sectional design of this study, and more research is needed in this area. Future studies should include a more diverse population and a larger sample size to enhance external validity. Additionally, no significant relationships were observed between water intake and PRAL, but a more acidifying diet pattern appeared to have an adverse influence on lean body mass. Our study highlights that the plant-to-animal protein ratio may be crucial in this relationship.

## Figures and Tables

**Table 1:** Inclusion and exclusion criteria for eligible participants for this study.

Inclusion Criteria	Exclusion Criteria
Women	Men
Aged ≥ 65 years	Cancer or dementia
Free of disease compromising 2-year survival	History of severe heart disease
Free and independently living	Organ failure (unstable, renal, respiratory, liver) or food allergy/intolerance necessitating a special diet
Competent to make own decisions	Diabetes mellitus type 1
	Diabetes mellitus type 2 with insulin therapy
	Chronic use of corticosteroid medication
	Change in habitual medication use

**Table 2:** Baseline characteristics of study participants according to tertiles of water intake and of PRAL.

Variable	Water Intake			*p* Value ^2^	PRAL			*p* Value ^2^
	T I (*n* = 65)	T II (*n* = 66)	T III (*n* = 64)		T I (*n* = 65)	T II (*n* = 65)	T III (*n* = 65)	
Age (y) ^1^	71.9 ± 3.7	72.7 ± 3.4	71.0 ± 3.3	0.263	72.2 ± 3.5	72.5 ± 3.5	71.8 ± 3.4	0.560
Education (y) ^1^	14.6 ± 2.3	14.5 ± 2.7	14.9 ± 3.0	0.040	14.8 ± 2.5	14.5 ± 2.9	14.8 ± 2.7	0.733
Marital status, *n* (%)								
Single	5 (8)	3 (4)	7 (11)	0.579	7 (11)	4 (6)	4 (6)	0.642
Married	28 (43)	23 (35)	21 (33)		26 (40)	23 (35)	23 (35)	
Divorced/separated	10 (15)	17 (26)	12 (19)		9 (14)	13 (20)	17 (26)	
widow	22 (34)	23 (35)	24 (37)		23 (35)	25 (39)	21 (33)	
Physical activity, *n* (%)								
Low	21 (32)	31 (47)	24 (38)	0.368	22 (34)	26 (40)	28 (43)	0.875
Moderate	38 (59)	28 (42)	31 (48)		35 (54)	32 (49)	30 (46)	
High	6 (9)	7 (11)	9 (14)		8 (12)	7 (11)	7 (11)	
Alcohol intake, *n* (%)								
Yes	54 (83)	56 (85)	50 (78)	0.587	58 (89)	53 (82)	49 (75)	0.120
No	11 (17)	10 (15)	14 (22)		7 (11)	12 (18)	16 (25)	
Current smoker, *n* (%)								
Yes	5 (8)	4 (6)	8 (12)	0.402	7 (11)	5 (8)	5 (8)	0.773
No	60 (92)	62 (94)	56 (88)		58 (89)	60 (92)	60 (92)	

^1^ Values are means ± SDs; ^2^ *p* values for comparisons of parameters between 3 groups tested by 1-factor ANOVA for the continuous variable or Pearson’s chi-square test for categorical variables; T-tertile.

**Table 3:** Relationship between tertiles of water intake and anthropometric measurements, body composition, and dietary characteristics ^1^.

Variable	Total (*n* = 195)	Water Intake			*p* Value ^2^
		T I (*n* = 65)	T II (*n* = 66)	T III (*n* = 64)	
Weight (kg)	72.5 ± 14.6	72.4 ± 13.8	73.5 ± 14.5	71.7 ± 15.6	0.577
Height (m)	1.6 ± 0.1	1.6 ± 0.1	1.6 ± 0.1	1.6 ± 0.1	0.662
BMI (kg/m^2^)	27.8 ± 4.6	28.1 ± 4.5	28.0 ± 4.7	27.2 ± 4.6	0.516
Android (%fat)	47.4 ± 8.7	48.9 ± 8.1	49.2 ± 6.9	44.2 ± 10.2	0.004
Gynoid (%fat)	49.0 ± 6.2	51.0 ± 5.7	49.5 ± 5.6	46.6 ± 6.6	<0.001
TBF (%fat)	42.8 ± 7.1	44.3 ± 6.9	43.7 ± 6.2	40.3 ± 7.7	0.005
Lean (kg)	38.3 ± 4.2	37.5 ± 3.4	38.0 ± 4.9	39.3 ± 4.0	0.021
ALM (kg/m^2^)	6.1 ± 0.9	6.1 ± 0.8	6.0 ± 1.0	6.3 ± 0.8	0.215
HGS (kg)	23.0 ± 4.8	22.9 ± 4.7	22.7 ± 4.2	23.5 ± 5.3	0.539
Water intake (mL /d)	2441.1 ± 577.7	1844.2 ± 257.8	2394.3 ± 139.7	3095.6 ± 367.1	<0.001
Energy intake (kcal/d)	1775.3 ± 518.9	1721.0 ± 544.3	1803.5 ± 527.8	1801.2 ± 486.1	0.375
Protein intake (g/d)	75.9 ± 22.4	72.6 ± 22.0	77.1 ± 21.8	78.1 ± 23.3	0.190
Fat intake (g/d)	68.5 ± 26.4	65.6 ± 26.3	69.6 ± 27.3	70.3 ± 25.8	0.523
Carbohydrates intake (g/d)	227.1 ± 74.2	223.0 ± 75.5	229.6 ± 79.6	228.6 ± 67.6	0.823
Fiber intake (g/d)	22.3 ± 8.0	20.4 ± 7.9	22.4 ± 8.0	24.1 ± 7.7	0.010
PRAL, mEq/d	0.9 ± 14.3	1.7 ± 12.5	1.5 ± 14.4	−0.6 ± 16.0	0.613

^1^ Values are means ± SDs; ^2^ *p* values for comparisons of parameters between 3 groups tested by 1-factor ANOVA for variables with normal distribution or Kruskal–Wallis test for variables with non-normal distribution; T-tertile; BMI—body mass index; TBF—total body fat; ALM—Appendicular Lean Mass index; HGS—hand grip strength; % fat—percent body fat; PRAL—potential renal acid load.

**Table 4:** Relationship between tertiles of PRAL and anthropometric measurements, body composition, and dietary characteristics ^1^.

Variable	PRAL			*p* Value ^2^
	T I (*n* = 65)	T II (*n* = 65)	T III (*n* = 65)	
Weight (kg)	69.1 ± 12.3	72.7 ± 14.8	75.8 ± 15.9	0.052
Height (m)	1.6 ± 0.1	1.6 ± 0.1	1.6 ± 0.1	0.358
BMI (kg/m^2^)	26.8 ± 3.7	28.1 ± 5.2	28.4 ± 4.6	0.087
Android (%fat)	47.1 ± 8.0	47.3 ± 10.0	47.9 ± 8.1	0.758
Gynoid (%fat)	48.1 ± 5.5	49.0 ± 7.0	50.0 ± 6.0	0.134
TBF (%fat)	42.1 ± 6.2	43.0 ± 8.2	43.3 ± 6.9	0.375
Lean (kg)	38.7 ± 4.2	38.3 ± 4.3	37.9 ± 4.2	0.505
ALM (kg/m^2^)	6.3 ± 0.8	6.1 ± 0.8	6.0 ± 1.1	0.127
HGS (kg)	23.2 ± 5.1	23.0 ± 4.7	22.8 ± 4.5	0.919
Water intake (mL/d)	2505.8 ± 561.9	2456.3 ± 683.8	2361.2 ± 466.1	0.494
Energy intake (kcal/d)	1745.2 ± 505.5	1640.7 ± 408.5	1939.9 ± 589.1	0.013
Protein intake (g/d)	69.5 ± 19.0	70.4 ± 15.8	87.9 ± 26.2	<0.001
Fat intake (g/d)	62.3 ± 25.5	64.4 ± 21.3	78.8 ± 29.1	<0.001
Carbohydrates intake (g/d)	242.3 ± 68.9	205.5 ± 56.6	233.4 ± 89.3	0.017
Fiber intake (g/d)	24.8 ± 7.2	20.4 ± 6.7	21.6 ± 9.2	0.001
PRAL, mEq/d	−14.6 ± 8.5	1.2 ± 3.0	15.9 ± 8.2	<0.001

^1^ Values are means ± SDs; ^2^ *p* values for comparisons of parameters between 3 groups tested by 1-factor ANOVA for variables with normal distribution or Kruskal–Wallis test for variables with non-normal distribution; T-tertile; BMI—body mass index; TBF—total body fat; ALM—Appendicular Lean Mass index; HGS—hand grip strength; % fat—percent body fat; PRAL—potential renal acid load.

**Table 5:** Spearman correlation coefficients between water intake or PRAL and body composition (r, *p*).

Variables	Water Intake		PRAL	
	Crude	Partial ^a^	Crude	Partial ^a^
Android (%fat)	−0.22 *p* = 0.002	−0.22 *p* = 0.001	0.04 *p* = 0.565	0.05 *p* = 0.526
Gynoid (%fat)	−0.29 *p* = 0.000	−0.29 *p* = 0.000	0.13 *p* = 0.065	0.13 *p* = 0.066
TBF (%fat)	−0.23 *p* = 0.001	−0.26 *p* = 0.002	0.08 *p* = 0.249	0.08 *p* = 0.252
Lean (kg)	0.19 *p* = 0.006	0.19 *p* = 0.007	−0.07 *p* = 0.304	−0.07 *p* = 0.312
ALM (kg/m^2^)	0.06 *p* = 0.379	0.05 *p* = 0.520	−0.17 *p* = 0.019	−0.16 *p* = 0.021
HGS (kg)	0.05 *p* = 0.507	0.05 *p* = 0.494	−0.05 *p* = 0.490	−0.06 *p* = 0.436

^a^ In contrast to the crude (not adjusted) the partial correlation coefficients were adjusted for age (years, continuous), physical activity (medium or high, low), living alone (yes, no), smoking (ever, never), alcohol consumption (ever, never), and medication use (no, yes).

**Table 6:** Characteristics of participants classified into two clusters ^1^.

Variable	Cluster		*p* Value ^2^
	Cluster 1 Low-PRAL Diet (*n* = 117)	Cluster 2 High-PRAL Diet (*n* = 78)	
Age (years)	72.2 ± 3.8	72.1 ± 3.0	0.896
Energy intake (kcal/d)	1545.9 ± 374.9	2119.3 ± 516.6	<0.001
Water intake (mL/d)	2432.8 ± 557.7	2453.5 ± 609.9	0.566
PP ratio	0.62 ± 0.24	0.44 ± 0.19	<0.001
PRAL, mEq/d	−5.7 ± 11.9	10.7 ± 11.8	<0.001
Protein intake (g/d)	63.3 ± 17.5	94.8 ± 19.7	<0.001
Fat intake (g/d)	57.0 ± 19.4	85.7 ± 26.4	<0.001
Carbohydrates intake (g/d)	207.2 ± 56.7	256.9 ± 86.7	<0.001
Fiber intake (g/d)	21.0 ± 7.0	24.1 ± 8.9	0.029
BMI (kg/m^2^)	27.3 ± 4.1	28.5 ± 5.1	0.192
Android (%fat)	47.5 ± 8.9	47.4 ± 8.6	0.892
Gynoid (%fat)	48.9 ± 6.3	49.2 ± 6.2	0.784
TBF (%fat)	42.7 ± 7.2	42.9 ± 7.0	0.979
Lean (kg)	38.5 ± 4.4	37.9 ± 3.8	0.263
ALM (kg/m^2^)	6.2 ± 0.8	6.0 ± 0.9	0.045
HGS (kg)	23.0 ± 5.0	23.0 ± 4.4	0.931

^1^ Values are means ± SDs; ^2^ *p* values for comparisons of parameters between 2 groups tested by 1-factor ANOVA for variables with normal distribution or Kruskal–Wallis test for variables with non-normal distribution; BMI—body mass index; TBF—total body fat; ALM—Appendicular Lean Mass index; HGS—hand grip strength; % fat—percent body fat; PRAL—potential renal acid load; PP—ratio of plant to animal protein.

## Data Availability

The original contributions presented in the study are included in the article/Supplementary Material, further inquiries can be directed to the corresponding author.

## Supplementary Materials

The following supporting information can be downloaded at: https://www.mdpi.com/article/10.3390/nu17111808/s1, Table S1. Characteristics of study participants according to cluster analysis; Figure S1. Mean values of the z-score of energy, water, PP ratio, PRAL, and protein in each cluster.
