# Supplementary material for: Water Intake, Dietary Acid Load, and Body Composition in Aging Females
Source: Nutrients. 2025 May 26;17(11):1808. doi: 10.3390/nu17111808 (PMC12158037; doi:10.3390/nu17111808)
Supplement: Supplementary file 1 [file nutrients-17-01808-s001.zip › nutrients-3641816-supplementary.pdf]

Table S1 Characteristics of study participants according to cluster analysis

Variable	Cluster 1 (n=117)	Cluster 2 (n=78)	p value[2]
Age (y) [1]	72.2±3.8	72.1±3.0	0.896
Education (y) [1]	14.4±2.4	15.1±3.0	0.089
Marital status, n (%)			
single	13 (11.1)	2 (2.6)	0.055
married	44 (37.6)	28 (35.9)	
divorced/separated	18 (15.4)	21 (26.9)	
widow	42 (35.9)	27 (34.6)	
Physical activity, n (%)			
low	45 (38.4)	31 (39.7)	0.425
moderate	56 (47.9)	41 (52.6)	
high	16 (13.7)	6 (7.7)	
Alcohol intake, n (%)			
yes	100 (85.5)	60 (76.9)	0.128
no	17 (14.5)	18 (23.1)	
Current smoker, n (%)			
yes	11 (9.4)	6 (7.7)	0.678
no	106 (90.6)	72 (92.3)	

[1]Values are means ± SDs; [2]P values for comparisons of parameters between 2 groups tested by 1-factor ANOVA for the continuous variable or Pearson's chi-square test for categorical variables.;

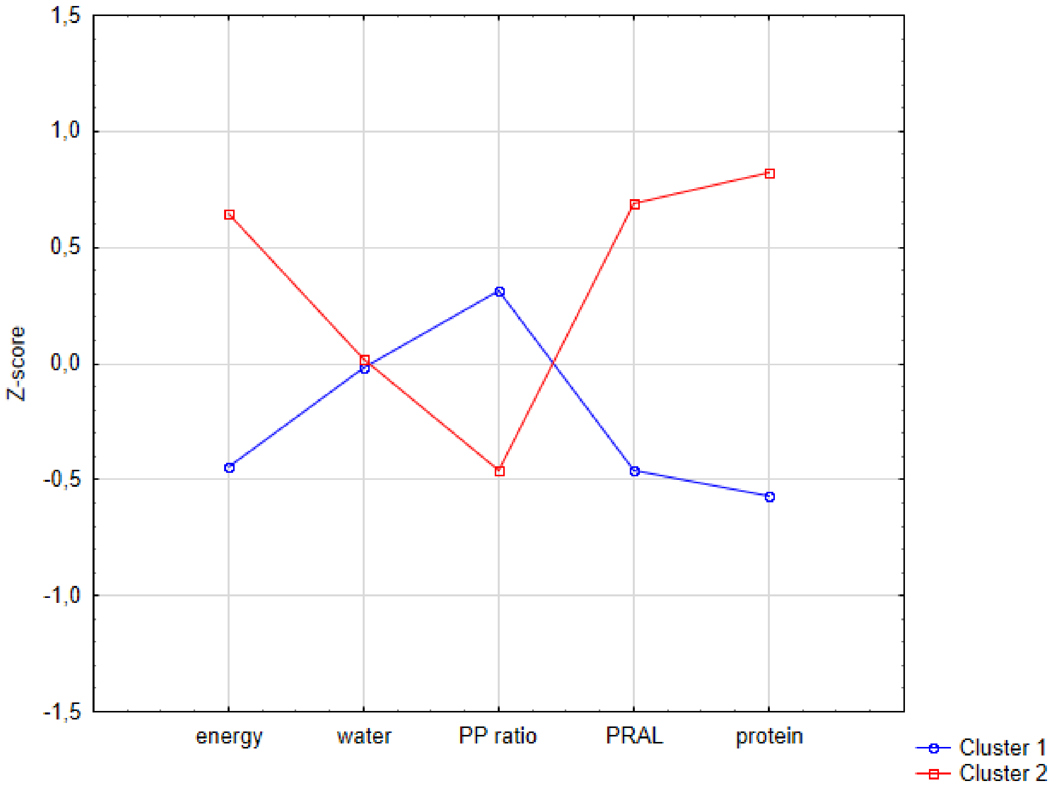

Figure S1. Mean values of the z-score of energy, water, PP ratio, PRAL and protein in each cluster
